# Ovarian Teratoma Masquerading as a CSF Pseudocyst in a Female with a Ventriculoperitoneal Shunt

**DOI:** 10.1155/2009/240705

**Published:** 2009-06-24

**Authors:** John M. K. Mislow, Jonathan R. Slotkin, Mark R. Proctor

**Affiliations:** ^1^Department of Neurosurgery, Children's Hospital of Boston and Brigham and Women's Hospital, Harvard Medical School, Boston, MA 02115, USA; ^2^Department of Neurosurgery, Children's Hospital of Boston, Harvard Medical School, Boston, MA 02115, USA

## Abstract

*Background*. In today's fast-paced and high-acuity emergency departments, clinicians are often compelled to triage cases so rapidly that a differential diagnosis consistent with the history and physical examination is not comprehensive. *Case Report*. This case report describes the unexpected finding of a cystic ovarian neoplasm in a young female with an abdominal mass and a ventriculoperitoneal shunt, initially diagnosed as a cerebrospinal fluid pseudocyst. We use this case to illustrate that the astute clinician must always synthesize a diagnosis from all data sources and not to rely on initial radiographic evaluations. *Conclusions*. This remarkable case demonstrates that all differential diagnoses must be entertained in order to rapidly and accurately diagnose a patient with a cystic abdominal mass.

## 1. Introduction

The modern Emergency Department is frequently a venue for fast-paced, high-acuity cases, and in many instances clinicians are compelled to limit their differential diagnosis due to time constraints. As an example, ventriculoperitoneal cerebrospinal fluid shunt failure is commonly the top differential diagnosis when a shunted patient presents with a wide variety of clinical problems—this is certainly a reasonable approach as long as it is not fixated on to the exclusion of all other appropriate differential diagnoses. The following case illustrates the pitfalls of such an oversimplification of differential diagnosis.

## 2. Case Report

**Figure 1:**
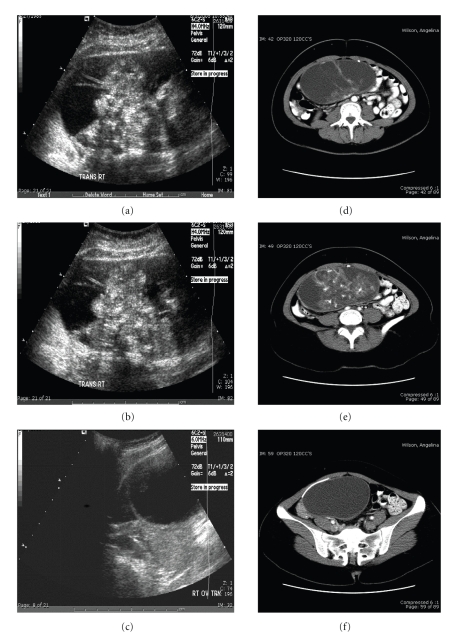
In figures (1a-1c), an abdominal ultrasound shows a large, walled, and multiseptated fluid collection. A small portion of the shunt catheter appears to be within this fluid collection, and thus the lesion was felt to be consistent with a pseudocyst, or CSFoma. In figures (1d-1f), a CT with IV contrast shows the same complex, multiseptated 16 × 8 × 17 cm pelvic mass emanating from the right ovary, with the distal shunt tubing draped circumferentially around the lesion, indicating that the shunt was not responsible. Surgical exploration leads to the diagnosis of teratoma.

A 21-year-old female with history significant for ventriculoperitoneal shunt-dependent hydrocephalus since infancy for myelomeningocele presented to our Emergency Department with an 8-day history of mild nausea and fevers up to 39°C. The patient had no history of shunt failure or revisions. She noted no neurological signs or symptoms, nor did she complain of a headache. The physical examination revealed a firm, nontender mass in the lower right abdominal quadrant. CT of head showed unremarkable ventricle size, good proximal shunt catheter placement, and no transependymal flow, although no previous imaging was available for comparison. An abdominal ultrasound was interpreted as a multiseptated fluid collection within the right lower quadrant extending into the lower abdomen, distinct from bowel, ovary, and uterus. A small portion of the shunt catheter was reported to be within this fluid collection; however; the tip could not be identified (Figure 1(a)–1(c)). As the patient had a history of a ventriculoperitoneal shunt with new onset of a cystic abdominal mass, the patient was diagnosed with a cerebrospinal fluid (CSF) pseudocyst, and the neurosurgical service was consulted. 

Because the tubing could not be localized within the mass, and the patient's history was not entirely consistent with a CSF pseudocyst (abdominal pain and distension, headaches, neurological deterioration) [1, 2], the neurosurgical service requested an abdominal CT. This study revealed a complex, multiseptated 16 × 8 × 17 cm pelvic mass emanating from the right ovary. The distal shunt tubing appeared to be draped circumferentially around the lesion and was not contained within the mass itself, as is generally the case with a CSF pseudocyst (Figure 1(d)–1(f)). This unexpected finding indicated that the shunt was independent of the lesion, and lead to an exhaustive differential diagnosis demonstrated in (Table 1) [3, 4]. Considering the patient's age, presentation, and sex, the differential was narrowed to abscess, seroma, mesenteric cyst, ovarian neoplasm, or pancreatic pseudocyst. Surgical exploration and resection of the lesion confirmed diagnosis of immature teratoma. As the teratoma was documented as stage I, no adjuvant therapy was required after resection of the lesion, and the patient remains disease-free to date. 

## 3. Discussion

 Ventriculoperitoneal shunt placement has proven to be an effective treatment of certain types of hydrocephalus; however, complications can occur. Unlike more common complications associated with ventriculoperitoneal shunts such as ventricular catheter obstruction, tubing disconnection, valve malfunction, and infection, peritoneal pseudocysts are a relatively rare complication. Originally described by Parry in 1975, ventriculoperitoneal CSF pseudocysts occur with an incidence ranging from 1% to 5% and are often associated with previous infections or revisions [1, 5–9]. Ascites can also present as a complication separate from pseudocysts [1] but is beyond the purview of this discussion. Pseudocyst formation, although most often occurring within weeks to a year after shunt placement, can occur late after shunting; in one report a pseudocyst was noted 10 years after ventriculoperitoneal shunt placement [10]. 

In the acute and fast-paced setting of the emergency ward, there is sometimes a tendency to suspect the CSF shunt system when a hydrocephalic patient presents with a wide variety of clinical problems. This is certainly a reasonable approach as long as it is not fixated on to the exclusion of all other appropriate differential diagnoses. In the setting of an abdominal mass, and with an initial ultrasound interpretation of a cyst in close proximity to the peritoneal tubing, the presumptive diagnosis of a CSF pseudocyst was quickly established. However, the history and physical examination of this patient were not consistent with this diagnosis. Accurate CT visualization of the patient's shunt system placed the abdominal tubing outside the cyst, and in conjunction with accurate anatomical correlation of the cyst and ovary, leads to the correct diagnosis and ultimately appropriate treatment.

## 4. Conclusions

This case illustrates the unexpected finding of a cystic ovarian neoplasm in a young female with a cystic abdominal mass in the setting of a ventriculoperitoneal shunt. Although accurate radiographic imaging and interpretations serve an invaluable role within medicine and surgery, clinicians must always rely on the foundation of an accurate and detailed history and physical examination to help guide them through all potential differential diagnoses in order to accurately diagnose and treat the patient.

## Figures and Tables

**Table 1:** Differential diagnosis of cystic abdominal masses in children (from Wooton-Gorges et al. [3]).

Liver	Mesenchymal hamartoma, Biloma, Parasitic cyst
Biliary system	Coledochal cyst, Hydrops of gallbladder
Spleen	Congenital cyst
Pancreas	Congenital cyst, Pseudocyst, Cystadenoma
Kidney/adrenal	Hydronephrosis, Multicystic dysplastic kidney, Multilocular cystic nephroma, Adrenal hemorrhage
Gastrointestinal	Mesenteric cyst/lymphangioma, Enteric/duplication cyst, Meconium pseudocyst
Genitourinary/ovary	Functional cyst, Teratoma/dermoid, Cystadenoma, Hematocolpos, Urachal cyst
Miscellaneous	Abscess, Teratoma, Necrotic or cystic changes in tumors CSF pseudocyst, Sacrococcygeal teratoma

## References

[1] de Oliveira RS, Barbosa A, de Andrade Vicente YAdeMV, Machado HR (2007). An alternative approach for management of abdominal cerebrospinal fluid pseudocysts in children. *Child's Nervous System*.

[2] Kariyattil R, Steinbok P, Singhal A, Cochrane DD (2007). Ascites and abdominal pseudocysts following ventriculoperitoneal shunt surgery: variations of the same theme. *Journal of Neurosurgery*.

[3] Wootton-Gorges SL, Thomas KB, Harned RK, Wu SR, Stein-Wexler R, Strain JD (2005). Giant cystic abdominal masses in children. *Pediatric Radiology*.

[4] Pernas JC, Catala J (2004). Case 72: pseudocyst around ventriculoperitoneal shunt. *Radiology*.

[5] Besson R, Hladky JP, Dhellemmes P, Debeugny P (1995). Peritoneal pseudocyst—ventriculo-peritoneal shunt complications. *European Journal of Pediatric Surgery*.

[6] Mobley LW, Doran SE, Hellbusch LC (2005). Abdominal pseudocyst: predisposing factors and treatment algorithm. *Pediatric Neurosurgery*.

[7] Parry SW, Schuhmacher JF, Llewellyn RC (1975). Abdominal pseudocysts and ascites formation after ventriculoperitoneal shunt procedures. Report of four cases. *Journal of Neurosurgery*.

[8] Rainov N, Schobeß A, Heidecke V, Burkert W (1994). Abdominal CSF pseudocysts in patients with ventriculo-peritoneal shunts. Report of fourteen cases and review of the literature. *Acta Neurochirurgica*.

[9] Roitberg BZ, Tomita T, McLone DG (1998). Abdominal cerebrospinal fluid pseudocyst: a complication of ventriculoperitoneal shunt in children. *Pediatric Neurosurgery*.

[10] Rovlias A, Kotsou S (2001). Giant abdominal CSF pseudocyst in an adult patient 10 years after a ventriculo-peritoneal shunt. *British Journal of Neurosurgery*.

